# Persistently elevated soluble MHC class I polypeptide-related sequence A and transforming growth factor-β1 levels are poor prognostic factors in head and neck squamous cell carcinoma after definitive chemoradiotherapy

**DOI:** 10.1371/journal.pone.0202224

**Published:** 2018-08-10

**Authors:** Jenny Ling-Yu Chen, Chien-Chung Chang, Yu-Sen Huang, Hung-Yang Kuo, Tzu-Yu Chen, Chun-Wei Wang, Sung-Hsin Kuo, Yu-Li Lin

**Affiliations:** 1 Division of Radiation Oncology, Department of Oncology, National Taiwan University Hospital and National Taiwan University College of Medicine, Taipei, Taiwan; 2 Institute of Biomedical Engineering, College of Medicine and College of Engineering, National Taiwan University, Taipei, Taiwan; 3 Department of Oncology, National Taiwan University Hospital Yun-Lin Branch, Yun-Lin, Taiwan; 4 Institute of Molecular and Cellular Biology, National Tsing-Hua University, Hsin-Chu, Taiwan; 5 Department of Medical Imaging, National Taiwan University Hospital and National Taiwan University College of Medicine, Taipei, Taiwan; 6 Department of Medical Research, National Taiwan University Hospital and National Taiwan University College of Medicine, Taipei, Taiwan; University of Cincinnati College of Medicine, UNITED STATES

## Abstract

We evaluated the prognostic significance of immunologic inhibitory biomarkers in head and neck squamous cell carcinoma (HNSCC) patients undergoing definitive chemoradiotherapy (CRT). Thirty patients were prospectively enrolled. Plasma levels of soluble MHC class I polypeptide-related sequence A (sMICA) and transforming growth factor-β1 (TGF-β1) were measured before and 2 weeks after CRT. The median follow-up was 32.9 months (range: 12.4–40.6 months). The pre-treatment sMICA (p < 0.001) and TGF-β1 (p < 0.001) levels were significantly increased in HNSCC patients, compared to healthy controls. In HNSCC patients, the median pre-CRT and post-CRT sMICA levels were 43.1 pg/mL and 65.3 pg/mL, respectively, while the median pre-CRT and post-CRT TGF-β1 levels were 57.7 ng/mL and 36.0 ng/mL, respectively. After CRT, 19 patients (63.3%) exhibited persistently elevated sMICA, six patients (20.0%) exhibited persistently elevated TGF-β1, and five patients (16.7%) exhibited persistently elevated sMICA and TGF-β1. Patients with persistently elevated sMICA and TGF-β1 after CRT experienced an earlier tumor progression (p = 0.030), and poor overall survival (p = 0.010). Our results suggest that HNSCC patients who exhibit persistently elevated sMICA and TGF-β1 levels after CRT are at higher risk of tumor progression or death.

## Introduction

Head and neck squamous cell carcinoma (HNSCC) is a highly aggressive solid tumor that may affect the nasal and oral cavities, pharynx, larynx, and paranasal sinuses. Modern definitive chemoradiotherapy (CRT) with curative intent is being increasingly used to treat HNSCC as it attempts to preserve organs. Identification of predictive plasma biomarkers of post-CRT response would serve as a simple and inexpensive means to determine patient risk early in the treatment and may facilitate individualized therapy [1].

Cancer patients are at high risk of impaired immune response, which would allow cancer cells to successfully escape immune surveillance [2]. One such mechanism involves soluble major histocompatibility complex (MHC) class I polypeptide-related sequence A (sMICA) [3]. MICA serves as a stress-induced ligand for the natural-killer group 2 member D (NKG2D) receptor and initiates the cytolytic responses of T cells and natural killer (NK) cells against cancer cells when bound to its receptor. By contrast, sMICA, which is produced by tumor cells, significantly blocks this binding and thus inhibits the antitumor activities of effector cells. Plasma sMICA concentration has previously been identified as a poor prognostic factor in numerous cancer types, including head and neck squamous cell carcinoma, hepatocellular carcinoma, multiple myeloma, non-small cell lung cancer, osteosarcoma, and prostate cancer [4–10]. Transforming growth factor-β1 (TGF-β1) is a multifunctional protein that regulates a variety of biological processes, including inflammation, tissue remodeling, development, and neoplastic transformation [11, 12]. TGF-β1 has also been identified as a stimulator of tumor invasion by inhibiting host immune activity [11, 13]. Theoretically, both sMICA and TGF-β1 markedly reduce tumor recognition by the immune system and largely impair the tumoricidal effects of NKG2D-dependent processes by diminishing NK cell-mediated tumoricidal activity [4]. We believe that the immunological inhibitory factors, sMICA and TGFβ, are of special interest and can act as potential biological prognostic factors.

Radiotherapy has been considered as a conventional strategy that benefits antitumor immunity [14]. The correlations of the immunosuppressive molecules sMICA and TGF-β1 and clinical survival outcomes after definitive chemoradiotherapy (CRT) have not been studied. Hence, we prospectively examined the plasma concentrations of sMICA and TGF-β1 before and after definitive CRT, to determine whether in patients with non-metastatic HNSCC receiving definitive CRT such a correlation exists.

## Materials and methods

### Patients

This study was approved by the Research Ethics Committee of the National Taiwan University Hospital (201706046RINC) and was performed in accordance with the Declaration of Helsinki, including all relevant details. The study is prospectively registered with ClinicalTrials.gov, number NCT03325036. Patients with newly diagnosed, non-metastatic HNSCC who underwent definitive CRT with curative intent between August 2014 and May 2015 were prospectively enrolled, irrespective of gender. Nasopharynx tumor was excluded due to its unique characteristics differing from other HNSCCs. Written informed consents were obtained from all patients. Tumors were staged according to the American Joint Commission on Cancer classification system, seventh edition[15]. The staging evaluations included magnetic resonance imaging (MRI) of the head and neck, chest radiography, liver ultrasonography, and bone scintigraphy, as well as positron emission tomography/computed tomography (CT) if clinically required. Histopathological grading was performed by an experienced pathologist. For patients with oropharyngeal SCC, immunohistochemistry was carried out for p16 on representative 4-μm sections cut from formalin-fixed, paraffin-embedded tissue blocks. P16 overexpression was defined as ≥ 70% cytoplasmic and nuclear staining [16]. A total of 14 age-matched healthy individuals (median age, 45 years; range, 33–60 years) served as healthy controls.

### Treatments

The radiotherapy treatment technique used in this study has been previously described [17]. Briefly, after the immobilization of the head using a thermoplastic mask, CT was performed with a 3–5-mm slice thickness of the head and neck region. The planning target volumes were evenly expanded using a 4-mm margin. Two dose fractionations (66 Gy in 33 fractions or 70 Gy in 33 fractions) were used, at the discretion of the treating physician. The plans were optimized using the inverse planning algorithm (Direct Machine Parameter Optimization) and heterogeneity corrections. All patients underwent intensity-modulated radiotherapy (IMRT), delivered by an Elekta Synergy accelerator (Elekta, Stockholm, Sweden) with a step-and-shoot technique. The treatment position was verified weekly using cone-beam CT X-ray volume imaging.

In most patients, the CRT regimen included 30 mg/m^2^ cisplatin weekly during radiotherapy. Two patients received taxane-based chemotherapies and one patient received tegafur-uracil alongside radiotherapy, at the discretion of the treating physician.

Head and neck specialist stipulated the need for neck dissection in patients with N2 or N3 disease of the neck, and with such dissection formally recommended to take place 4 to 8 weeks after the completion of CRT. In our study, neck dissections were performed in 27% of patients after completion of CRT.

### Quantification of sMICA and TGF-β1 in patients with HNSCC

Plasma samples were collected before and 2 weeks after definitive CRT. At each time point, a 10-ml sample of blood was drawn from the patients, placed in ethylene-diamine-tetra-acetic acid (EDTA) tubes, and centrifuged at 1140 g (2500 rpm) for 30 min within 30–40 min of collection. Plasma was divided into aliquots and stored aseptically at −80°C until analysis. Quantitative human ELISA kits for sMICA and TGF-β1 (R&D Systems) were used to measure each biomarker in plasma samples. The absolute levels of each biomarker were compared in the before and after CRT plasma samples.

### Follow-up

Follow-up analyses were conducted using a comprehensive protocol with the relevant data available on February 15, 2018. Follow-up visits were conducted at 1, 2, 3, 6, 9, and 12 months post-CRT and every 3 months thereafter. Treatment response after CRT was assessed every 3 months using endoscopy and head and neck MRI as well as biopsy of suspicious lesions, and the best overall response recorded from completion of CRT until disease progression was registered. Chest radiographs were taken every 6 months, whereas CT, MRI, bone scanning, or other investigations were performed when there was clinical suspicion of tumor progression. Tumor progression analysis considered locoregional recurrence (LRR) and distant metastasis (DM), determined by physicians and radiologists via a panel discussion. LRR was defined as recurrence at the head and neck region or the cervical nodal sites and DM as metastasis beyond the locoregional sites based on pathologic, cytologic, or radiologic evidence. Histological confirmation was not mandatory for LRR or DM. Patients who had no tumor progression at the time of analysis were censored at the date of last contact. Progression-free survival (PFS) was defined as the time (months) from the date of completed treatment to that of disease progression or censoring. Overall survival (OS) was the time (months) from the date of completed treatment to that of death or censoring.

### Statistical analysis

The statistical analysis was performed using SPSS for Windows, version 17.0 (SPSS, Chicago, IL, USA). Statistical comparisons of pre-treatment sMICA or TGF-β1 levels between patients or healthy controls were performed using the nonparametric Mann-Whitney U test. Statistical comparisons between sMICA or TGF-β1 levels before and after CRT were performed by nonparametric Wilcoxon rank test. Statistical comparisons between sMICA or TGF-β1 levels and clinical factors (age, stage groupings, site, T stage, N stage, histologic grade, or best overall response registered) were performed by nonparametric Mann-Whitney U test. The analysis was conducted using follow-up data available on February 1, 2018. Kaplan–Meier life table analysis and the log-rank test were used to assess survival times and to compare survival according to prognostic factors. A *p*-value of < 0.05 was considered statistically significant. All prognostic variables found to be significant in the univariate analysis were included in a multivariate analysis based on the Cox proportional hazards regression model. At post hoc analysis, the sample size of 30 patients provides 94% power to detect a difference in the median survival of 14.0 months versus 30.0 months, assuming 10 months of accrual and 32.9 months of follow-up in a two-sided log-rank test with an α level of 0.05.

## Results

### Patient characteristics

Thirty non-metastatic HNSCC patients were included in this study (median age, 55 years; range, 32–70 years) (Table 1). The primary tumor sites were the oropharynx (36.7%), hypopharynx (26.7%), larynx (20.0%), oral cavity (13.3%), and others (3.3%). In oropharyngeal cancer patients, 64% overexpressed p16 as observed on immunohistochemistry. Most patients (73.4%) had stage IV disease. Five patients with stage I-II HNSCC who were either considered poor surgical candidates on the basis of pre-existing medical conditions or refused to undergo surgery were also included (two oropharynx, two hypopharynx, and one larynx by tumor sites). The median radiotherapy dose was 70 Gy in 33 fractions (66–70 Gy in 33 fractions). Most patients (90%) received weekly cisplatin with a median of 6 cycles during definitive CRT (median cisplatin cumulative dose: 180 mg/m^2^, range: 120–210 mg/m^2^). Other concurrent chemotherapy regimens include taxane-based chemotherapies (n = 2) and tegafur-uracil (n = 1), at the discretion of the treating physician. Neck dissections were performed in 27% of patients at a median of 5 weeks (range, 4–8 weeks) post CRT. The best overall response registered was complete response (46.7%), partial response (33.3%), stable disease (6.7%), or progressive disease (13.3%).

**Table 1:** Clinical characteristics and treatment parameters for the enrolled HNSCC patients (n = 30).

	Total (n = 30)
Age	
median (range)	55 (32–70)
Gender	
Male / Female	30 / 0
Smoking history, n (%)	
Yes	17 (56.7)
No	13 (43.3)
Tumor site, n (%)	
Oral cavity	4 (13.3)
Oropharynx	11 (36.7)
Hypopharynx	8 (26.7)
Larynx	6 (20.0)
Unknown primary	1 (3.3)
Clinical T stage ^a^, n (%)	
T1	4 (13.4)
T2	10 (33.3)
T3	3 (10.0)
T4	13 (43.3)
Clinical N stage ^a^, n (%)	
N0	5 (16.7)
N1	8 (26.6)
N2	12 (40.0)
N3	5 (16.7)
Stage groupings ^a^, n (%)	
I	1 (3.3)
II	4 (13.3)
III	3 (10.0)
IVA	17 (56.7)
IVB	5 (16.7)
Histologic grade, n (%)	
Low (well differentiated)	2 (6.7)
Intermediate (moderately differentiated)	18 (60.0)
High (poorly differentiated, undifferentiated)	10 (33.3)
Radiotherapy total dose, n (%)	
≥ 70 Gy	20 (66.7)
≤ 66 Gy	10 (33.3)
Concurrent systemic therapy, n (%)	
Weekly cisplatin	27 (90.0)
Other regimens ^b^	3 (10.0)
Cumulative cisplatin dose, n (%)	
≥ 200 mg/m^2^	5 (16.7)
< 200 mg/m^2^	25 (83.3)
Neck dissection post CRT ^c^, n (%)	
Preformed	8 (26.6)
Not performed	22 (73.4)

*Abbreviations*: HNSCC = head and neck squamous cell carcinoma. CRT = chemoradiotherapy.

^a^ Stage is classified by the American Joint Committee on Cancer 7th edition.

^b^ Other regimens include taxane-based chemotherapies (n = 2) and tegafur-uracil (n = 1).

^c^ Neck dissections were performed at a median of 5 weeks (range, 4–8 weeks) post CRT.

During a median follow-up of 32.9 months (range: 12.4–40.6 months), tumor progression was noted in 15 patients (50.0%) with five LRR, nine DM, and one both LRR and DM cases, registered by first tumor progression site. Eleven patients (36.7%) died.

### Association of plasma immunologic inhibitory biomarkers with clinical outcomes

**Fig 1:**
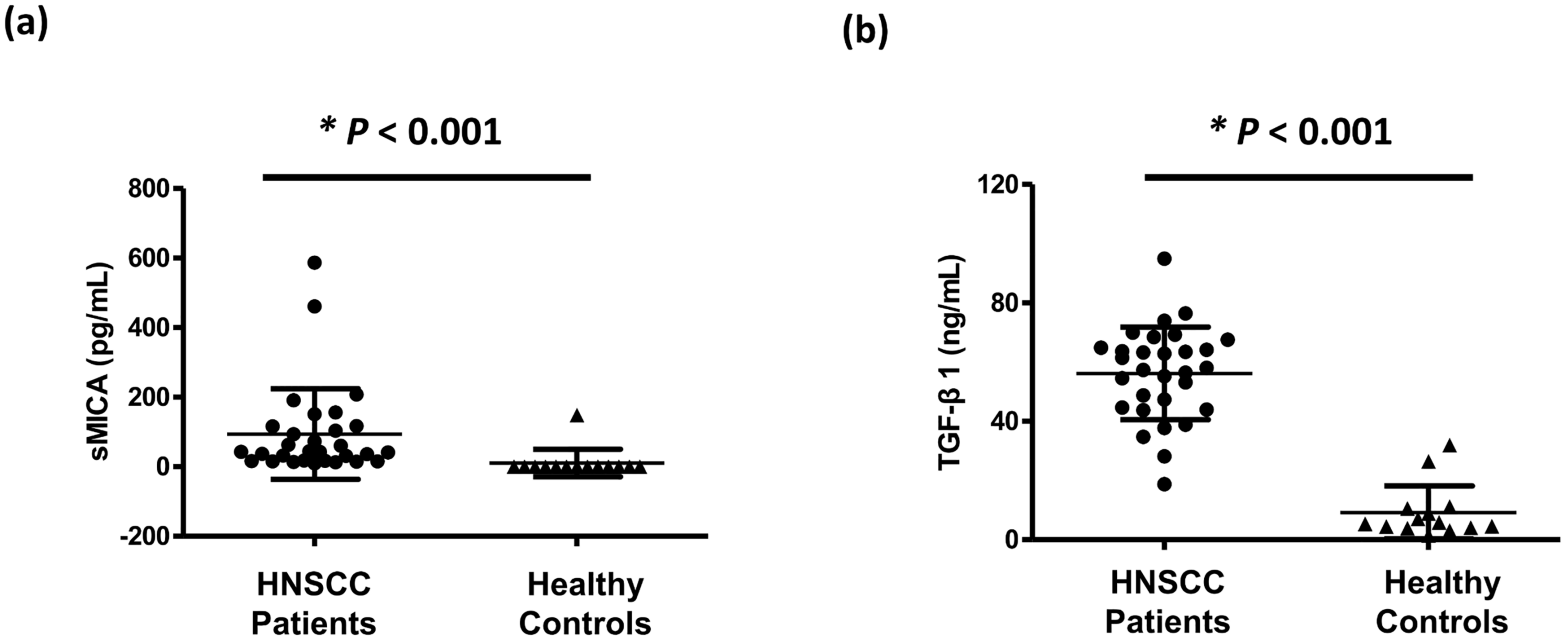
Plasma concentrations of soluble MHC class I polypeptide-related sequence A (sMICA) and transforming growth factor (TGF)-β1 between head and neck squamous cell carcinoma (HNSCC) patients and healthy controls. Mean and standard deviation of pre-treatment levels of plasma (a) sMICA and (b) TGF-β1 between patients and healthy controls. P-values for statistical comparisons of pre-treatment sMICA or TGF-β1 levels between patients or healthy controls were performed by nonparametric Mann-Whitney U test.

The plasma levels of sMICA and TGF-β1 were quantified before and at 2 weeks after CRT. The pre-treatment sMICA levels (median, 43.1 pg/mL in HNSCC patients *vs* 0.0 pg/mL in healthy controls, p < 0.001) and TGF-β1 levels (median, 57.7 ng/mL in HNSCC patients *vs* 5.6 ng/mL in healthy controls, p < 0.001) were significantly elevated in HNSCC patients compared to healthy individuals (Fig 1).

**Fig 2:**
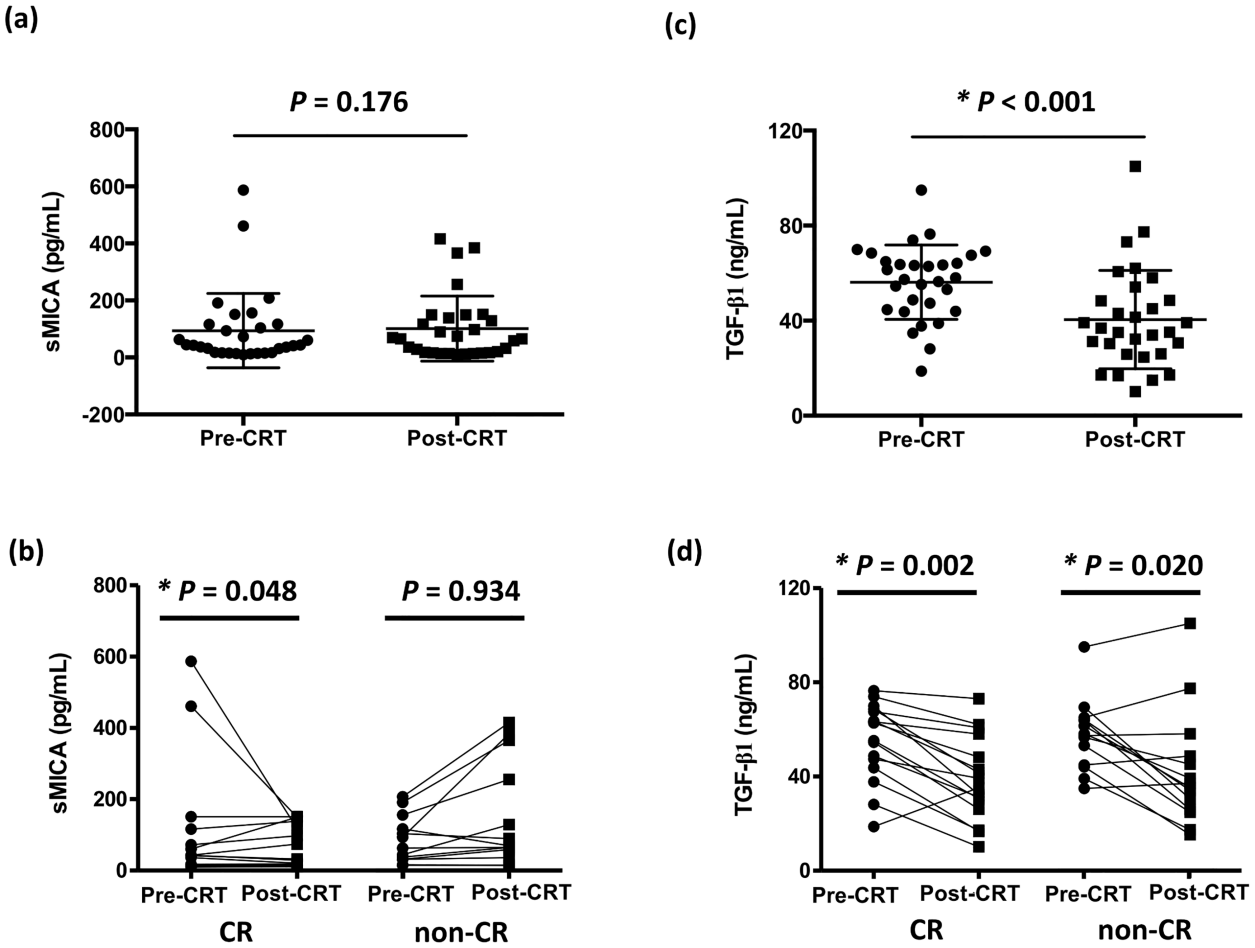
Plasma concentrations of soluble MHC class I polypeptide-related sequence A (sMICA) and transforming growth factor (TGF)-β1 pre- and post-chemoradiotherapy (CRT). Mean and standard deviation of pre- and post-CRT levels of plasma (a) sMICA and (c) TGF-β1 in all patients. Mean and standard deviation of pre- and post-CRT levels of plasma (b) sMICA and (d) TGF-β1 in patients who achieved complete response (CR) and in patients who did not. P-values for statistical comparisons between sMICA or TGF-β1 levels before and after CRT were performed by nonparametric Wilcoxon rank test.

Among all patients, the pre-CRT and post-CRT sMICA levels ranged from 9.7 to 587.2 pg/mL (media, 43.1 pg/mL) and from 10.8 to 416.1 pg/mL (median, 65.3 pg/mL), respectively (Fig 2a). No statistically significant difference was seen between sMICA before and after CRT by nonparametric Wilcoxon rank test (p = 0.176) when including all patients as a single cohort. When stratifying patients by their best response after treatment (Fig 2b), patients who achieved CR had significantly lower post-CRT sMICA levels (p = 0.048), indicating the value of sMICA as prognostic biomarker. As shown in Table 2, the pre-CRT sMICA level was not related to clinical T stage (p = 0.650), clinical N stage (p = 0.152), stage groupings (p = 0.256), tumor site (p = 0.237), p16 overexpression (p = 0.131), age (p = 0.271), or best overall response registered (p = 0.591). The post-CRT sMICA levels was also not related to best overall response (p = 0.714). The numerical values of sMICA levels in HNSCC patients grouped by stage, site, or p16 overexpression are presented in S1 Fig.

**Table 2:** Relationships of sMICA and TGF-β1 levels and clinical features of enrolled HNSCC patients.

	p-value ^a^ (n = 30)	
	Pre-CRT sMICA level	Pre-CRT TGF-β1 level
Age ≥ 60 years	0.271	0.345
Stage ≥ IV	0.256	0.348
Site (Non-oropharynx)	0.237	0.621
Oropharynx SCC (non-p16 overexpression *vs* p16 overexpression)^b^	0.131	0.345
Clinical T4 stage	0.650	0.464
Clinical N3 stage	0.152	0.253
High histologic grade	0.836	0.344
Best overall response registered (non-CR *vs* CR)	0.591	0.914

*Abbreviations*: HNSCC: head and neck squamous cell carcinoma; CRT: Chemoradiotherapy; sMICA: soluble MHC class I polypeptide-related sequence A; TGF-β1: transforming growth factor-β1; CR: complete response.

^a^ Significance tested using nonparametric Mann-Whitney U test.

^b^ In eleven oropharyngeal cancer patients, seven (64%) had p16 overexpression based on immunohistochemistry.

Among all patients, as seen in Fig 2c, the pre-CRT and post-CRT TGF-β1 levels ranged from 18.8 to 95.0 ng/mL (median, 57.7 ng/mL) and from 10.1 to 105.0 ng/mL (median, 36.0 ng/mL), respectively. Statistically significant difference was seen between TGF-β1 before and after CRT by nonparametric Wilcoxon rank test (p < 0.001). When stratifying patients by their best response after treatment, as shown in Fig 2d, statistically significant difference could still be observed in TGF-β1 levels before and after CRT in patients who had achieved CR and those who had not. As shown in Table 2, the pre-CRT TGF-β1 level was not related to clinical T stage (p = 0.464), clinical N stage (p = 0.253), stage groupings (p = 0.348), tumor site (p = 0.621), age (p = 0.345), or best overall response registered (p = 0.914). The post-CRT TGF-β1 levels was also not related to best overall response registered (p = 0.395). The numerical values of TGF-β1 levels in HNSCC patients grouped by stage, site, or p16 overexpression are presented in S1 Fig.

### Predictors of tumor progression in patients with HNSCC

Persistently elevated levels of the biomarkers were defined as levels after CRT being greater than or equal to the levels before CRT. In the study cohort, 19 patients (63.3%) exhibited persistently elevated sMICA after CRT, six patients (20.0%) exhibited persistently elevated TGF-β1 after CRT, and five patients (16.7%) exhibited persistently elevated sMICA and TGF-β1 after CRT. In patients with persistently elevated sMICA (n = 19), the median elevation in sMICA level (the difference between pre-CRT and post-CRT levels) was 26.0 pg/mL (range: 1.1–291.5 pg/mL). In patients with persistently elevated TGF-β1 (n = 6), the median elevation in TGF-β1 level was 6.9 ng/mL (range: 0.8–16.4 ng/mL).

**Fig 3:**
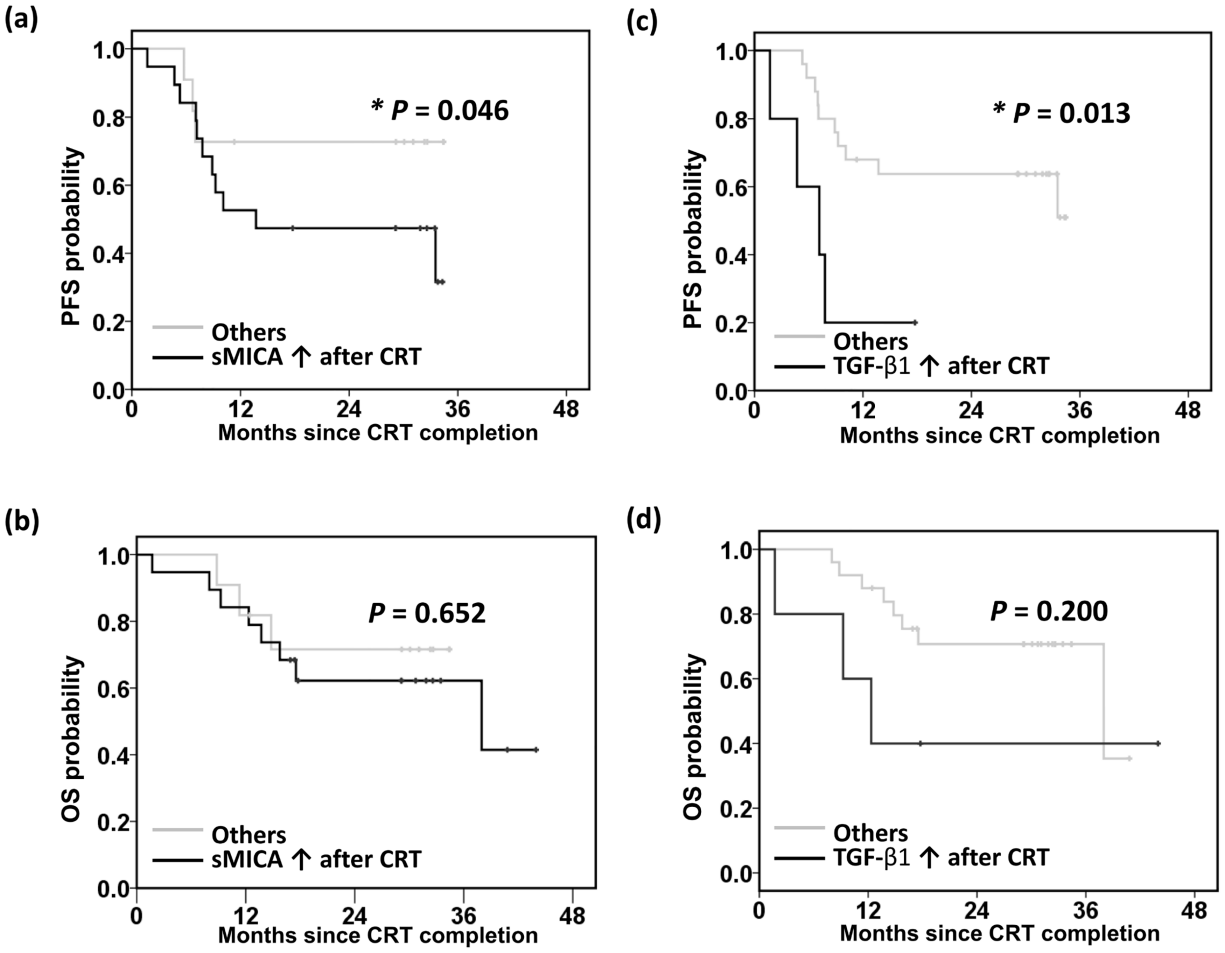
Clinical outcomes of 30 head and neck squamous cell carcinoma patients receiving definitive chemoradiotherapy (CRT). Patients were stratified according to whether they did or did not exhibit persistently elevated levels of soluble MHC class I polypeptide-related sequence A (sMICA) or transforming growth factor (TGF)-β1 after CRT. The groups were then compared with respect to (a, c) progression-free survival (PFS), and (b, d) overall survival (OS). Persistently elevated sMICA (p = 0.046) or TGF-β1 (p = 0.013) after CRT correlated with an earlier tumor progression. p-values were determined using Kaplan–Meier log-rank tests.

To identify the importance of sMICA and TGF-β1 in tumor progression and survival after CRT, patients were grouped according to the presence or absence of persistently elevated sMICA and TGF-β1 levels after CRT. Compared to patients with decreased sMICA after CRT, patients with persistently elevated sMICA after CRT experienced an earlier tumor progression (mean PFS survival of 20.0 months vs 26.8 months, p = 0.046, Fig 3a); however, the overall survival difference was not statistically significance (mean overall survival of 27.9 months vs 30.4 months, p = 0.652, Fig 3b). Compared to patients with decreased TGF-β1 after CRT, patients with persistently elevated TGF-β1 after CRT experienced an earlier tumor progression (mean PFS survival of 7.8 months vs 24.8 months, p = 0.013, Fig 3c), and overall survival difference was not statistically significance (mean overall survival of 22.2 months vs 31.6 months, p = 0.200, Fig 3d).

**Fig 4:**
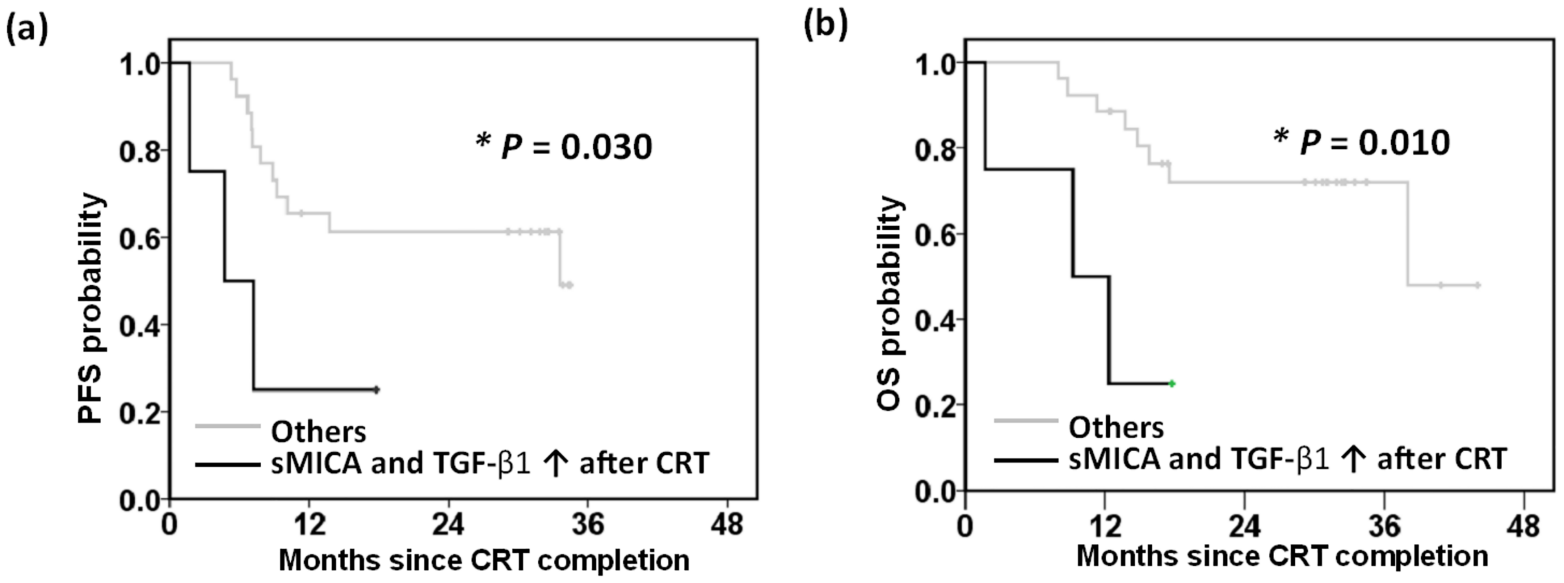
Patients with persistently elevated soluble MHC class I polypeptide-related sequence A (sMICA) and transforming growth factor (TGF)-β1 after chemoradiotherapy (CRT) experienced an earlier tumor progression and poor overall survival. Patients were stratified according to whether they did or did not exhibit persistently elevated levels of sMICA and TGF-β1 after CRT. The groups were then compared with respect to (a) progression-free survival (PFS), and (b) overall survival (OS). Persistently elevated sMICA and TGF-β1 levels after CRT correlated with an earlier tumor progression (p = 0.030) and poor overall survival (p = 0.010). p-values were determined using Kaplan–Meier log-rank tests.

We hypothesized that due to the inhibitory role of sMICA and TGF-β1, their expression can be used as a prognostic biomarker, with persistently elevated sMICA and TGF-β1 levels after CRT being identified as a potential prognostic factor. Patients with persistently elevated sMICA and TGF-β1 after CRT experienced an earlier tumor progression (mean PFS survival of 7.8 months vs 24.2 months, p = 0.030, Fig 4a), and poor overall survival (mean overall survival of 10.3 months vs 33.8 months, p = 0.010, Fig 4b).

In a univariate analysis (Table 3), clinical T4 disease (p = 0.007), stage ≥ IV (p = 0.031), and persistently high sMICA and TGF-β1 levels (p = 0.030) were significantly associated with tumor progression; these factors were significantly associated with death. In a multivariate analysis (Table 3), none of the prognostic factors reached statistical significance in predicting tumor progression, and clinical T4 stage remained the significant factor in predicting death (hazard ratio 4.9, 95% confidence interval 1.3–18.7, p = 0.020).

**Table 3:** Univariate and multivariate analyses of potential prognostic factors for survival in enrolled HNSCC patients.

(n = 30)	Progression-free survival		Overall survival	
	Univariate analysis	Multivariate analysis	Univariate analysis	Multivariate analysis p-value
	p-value ^a^	HR (95% CI), p-value ^b^	p-value ^a^	HR (95% CI), p-value ^b^
Age ≥ 60 years	0.132		0.644	
Stage ≥ IV	0.031	3.3 (0.4–30.8), p = 0.286	0.026	8.4 (0.0–17.9), p = 0.966
Site (Non-oropharynx)	0.494		0.331	
Clinical T4 stage	0.007	2.5 (0.8–8.1), p = 0.136	0.009	4.9 (1.3–18.7), p = 0.020
Clinical N3 stage	0.323		0.267	
High histologic grade	0.262		0.104	
Cumulative cisplatin dose < 200 mg/m^2^	0.130		0.113	
Radiotherapy total dose ≤ 66 Gy	0.548		0.877	
Persistently elevated sMICA and TGF-β1 ^c^	0.030	2.7 (0.8–9.1), p = 0.100	0.010	1.5 (0.3–7.7), p = 0.651

*Abbreviations*: HNSCC: head and neck squamous cell carcinoma; CRT: Chemoradiotherapy; sMICA: soluble MHC class I polypeptide-related sequence A; TGF-β1: transforming growth factor-β1.

^a^ Significance tested using Kaplan–Meier life table analysis and the log-rank test.

^b^ Significance tested using multivariate analysis based on the Cox proportional hazards regression model.

^c^ Persistently elevated levels of factors were defined as below: levels after CRT were greater than or equal to levels before CRT.

## Discussion

To date, this was the first study to address the association of plasma immunologic inhibition biomarkers with clinical outcomes in patients with non-metastatic HNSCC receiving definitive CRT. We found that after definitive CRT treatment, persistently elevated sMICA and TGF-β1 levels were associated with poor clinical outcomes. Impaired immune surveillance, mediated by immune escaping mechanisms of tumors, may be the main cause of tumor progression in patients with HNSCC [2]. Radiotherapy would overcome the obstacles of tumor immune escaping, by inducing cancer cell death; the subsequent release of antigens in association with pro-inflammatory signals triggers the innate immune system to activate tumor-specific T cells and enhance the infiltration of activated T cells into the tumor microenvironment [14]. In patients without tumor progression in our study, the decreased levels of plasma immunologic inhibitory biomarkers, including sMICA and TGF-β1 post-CRT, indicated that radiotherapy could have overcome the barriers to impaired immune surveillance and tumor immune escaping.

Tumor-specific stress ligands that inhibit effector cells can serve as adequate molecular markers of solid tumor progression [18]. Tumor shedding of NKGD ligands (i.e. sMICA) provide a means by which the tumor can escape immune surveillance; the predominant form of this type of escape involves reduced NKG2D expression on the surface of circulating blood lymphocytes (e.g. NK and CD8+ T cells), resulting in attenuated recognition of malignant cancer cells and reduced tumor surveillance [19]. Plasma sMICA concentration has also been associated with poor prognosis in hepatocellular carcinoma, multiple myeloma, non-small cell lung cancer, and osteosarcoma. In all these studies the elevated sMICA level enhanced tumor progression, by deteriorating NK cell-mediated cytotoxicity and enhancing tumor evasion from immune surveillance [4–10]. Kumar et al demonstrated that sMICA was significantly elevated in HBV-induced hepatocellular carcinoma, and patients with sMICA levels >5 pg/mL exhibited poorer prognosis than those with serum sMICA levels below this threshold [5]. Wang et al reported that serum sMICA levels were significantly higher in non-small cell lung cancer patients than in healthy controls, and high levels of sMICA are an independent predictor for metastasis and survival [9]. Rebmann et al also revealed sMICA as an independent predictive factor for overall and progression-free survival in multiple myeloma patients [7].

In our study, instead of setting a cut-off point for sMICA plasma levels, we considered persistently elevated sMICA and TGF-β1 after CRT as poor prognostic factor, as it resulted in less inter-subject variability when analyzing the paired data before and after treatment, resulting in more precise comparisons with fewer subjects [20]. Our study demonstrated that the pre-treatment sMICA and TGF-β1 plasma levels were markedly elevated in HNSCC patients when compared to healthy controls, and patients with persistently elevated sMICA and TGF-β1 levels after treatment had a higher tumor progression rate. Our findings are in line with the study by Kloss et al, which reported a difference in the plasma concentrations of sMICA and TGF-β1 in healthy controls versus patients. They also suggested that elevated levels of sMICA and TGF-β1 concentrations correlates with tumor progression[4]. Based on the above information, sMICA and TGF-β1 are potent prognostic markers associated with higher risk of tumor progression.

When addressing tumor immune escaping, TGF-β1 reduced NK cell-mediated killing activity and decreased NKG2D expression [4]. TGF-β1 reportedly downregulated the expression of NKG2D and CD16 on NK cells and inhibited the biological functions of these cells in a murine HNSCC model [21]. In patients with HNSCC, elevated post-radiotherapy levels of TGF-β1 were found to correlate with a poorer prognosis [22]. Patients in our study with high post-CRT plasma TGF-β1 levels were more prone to tumor progression, which agrees with previous results [21, 22], showing that post-CRT TGF-β1 levels indicate tumor immune escaping, leading to tumor progression.

Radiotherapy, as a complementary method in priming and effector phases of antitumor immunity, is an appealing strategy for generation of immunity against the patient’s self-tumor. The activation of immunological memory can result in long-lasting systemic responses [23]. Clinical cohort studies have demonstrated the efficacy of radiotherapy when combined with available immunotherapies [24, 25]. In patients with advanced non-small-cell lung cancer, a history of radiotherapy is associated with longer survival in response to pembrolizumab, compared to a lack of previous radiotherapy, further suggesting that the optimal treatment strategy should combine radiotherapy and immunotherapy [26].

Our institution exercises strict policies on post-CRT neck dissection in patients with initial N2 or N3 disease of the neck. The post-CRT neck dissection rate was 26.6% in the present study, which is close to the 31–35% rate described by Cohen et al [27]. In our study cohort, half of the patients experienced tumor progression. The failure rate was higher than expected, which could be contributed to the higher prevalence (73.4%) of pharyngeal carcinoma in our study, alongside inadequate cumulative cisplatin dosage. Patients with pharyngeal primary carcinoma reportedly have a higher risk of tumor recurrence, whereas patients with hypopharyngeal cancers may be prone to distant metastases [28]. Concomitant weekly cisplatin (30 mg/m^2^ per week) does not appear to be superior to triweekly cisplatin in improving oncological outcomes and decreasing early effects of treatment in head and neck cancer patients, since the cumulative cisplatin dose in a weekly schedule does not reach the 200mg/m^2^ threshold [29, 30]. In the present study, patients with cumulative cisplatin dose < 200 mg/m^2^ followed a trend toward tumor progression (p = 0.130) or death (p = 0.113). Although our patient enrollment process did not discriminate for gender, our study eventually included thirty male patients and no female patients. The phenomenon that only male patients were included in the study would be explained by the epidemiology of HNSCC in Taiwan, which occurs predominantly in men, with a male to female ratio ranging from 6:1 in the cancers of the oral cavity to 34:1 in the cancers of the hypopharynx [31].

This study had a few limitations. The relatively small number of patients might have led us to over-estimate the significance of the immunologic inhibitory biomarkers. We believe the results should be validated in a larger independent cohort. The heterogeneous origin of tumors (oropharynx, hypopharynx, larynx, or oral cavity) may further confound the tumor progression patterns. The fact that only males were enrolled in the present study might limit the use of the outcomes from this study. Nevertheless, the analysis of immunologic inhibitory biomarkers might give us an insight into the course of the disease. For patients predicted to have a poor prognosis, more intensive therapy after CRT including adjuvant chemotherapy or adjuvant immunologic therapy should be considered. In conclusion, our results suggest that persistently elevated sMICA and TGF-β1 levels after CRT are associated with higher risk of tumor progression and poor overall survival.

## Supporting information

S1 Fig Pre-treatment plasma concentrations of soluble MHC class I polypeptide-related sequence A (sMICA) and transforming growth factor (TGF)-β1 in head and neck squamous cell carcinoma (HNSCC) patients. Mean and standard deviation of pre-treatment levels of plasma (a,b,c) sMICA and (d, e, f) TGF-β1 in HNSCC patients according to stage, site, or p16 overexpression. P-values for statistical comparisons of pre-treatment sMICA or TGF-β1 levels between groups were calculated using the nonparametric Mann-Whitney U test.
(TIF)
