# Supplementary figures and images for: Persistently elevated soluble MHC class I polypeptide-related sequence A and transforming growth factor-β1 levels are poor prognostic factors in head and neck squamous cell carcinoma after definitive chemoradiotherapy
Source: PLoS One. 2018 Aug 10;13(8):e0202224. doi: 10.1371/journal.pone.0202224 (PMC6086445; doi:10.1371/journal.pone.0202224)

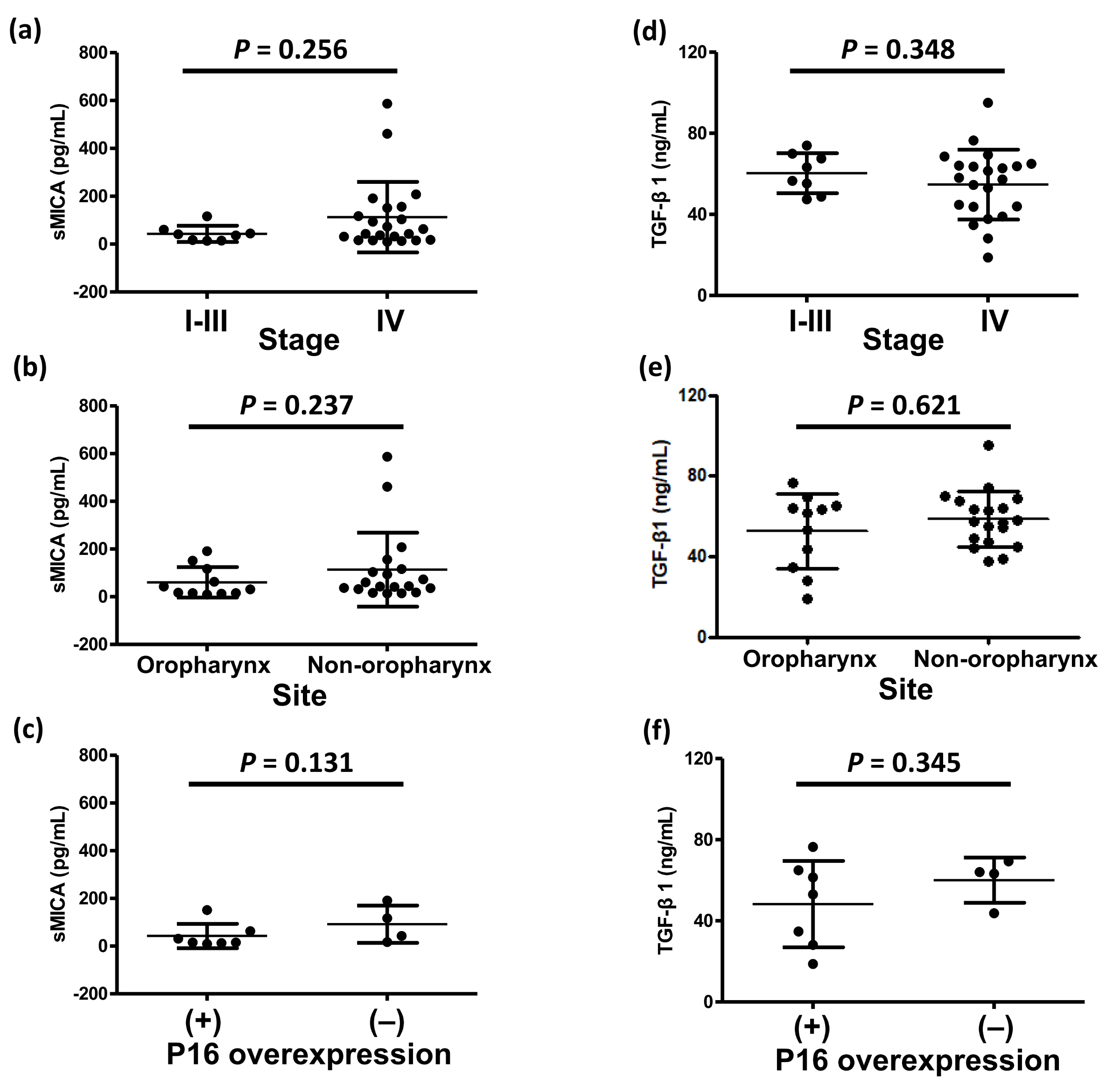

Supplement: S1 Fig — Mean and standard deviation of pre-treatment levels of plasma (a,b,c) sMICA and (d, e, f) TGF-β1 in HNSCC patients according to stage, site, or p16 overexpression. P-values for statistical comparisons of pre-treatment sMICA or TGF-β1 levels between groups were calculated using the nonparametric Mann-Whitney U test. (TIF) [file pone.0202224.s001.tif]
